# Brugada Syndrome: Evolving Insights and Emerging Treatment Strategies

**DOI:** 10.19102/icrm.2017.080205

**Published:** 2017-02-15

**Authors:** Stephen A. Duffett, Jason D. Roberts

**Affiliations:** ^1^Section of Cardiac Electrophysiology, Division of Cardiology, Department of Medicine, Western University, London, Ontario, Canada

**Keywords:** Brugada syndrome, catheter ablation, genetics, sudden cardiac death

## Abstract

Brugada syndrome (BrS) is a rare inherited arrhythmia disorder associated with sudden cardiac death secondary to malignant ventricular arrhythmias. Since its first mention approximately 25 years ago, major strides have been made towards unraveling the condition’s genetic and mechanistic underpinnings. Despite considerable progress, however, gaps in the understanding of BrS continue to persist, and clinical management of affected individuals remains challenging. Identification of an underlying genetic culprit continues to be elusive in the majority of patients, while discord regarding the condition’s underlying pathophysiology also persists, with strong lines of evidence present for both the “depolarization” and “repolarization” hypotheses. Exciting new therapeutic options hold significant promise, including substrate-based catheter ablation and the subcutaneous implantable cardioverter-defibrillator, although the decision of when to intervene in the cases of asymptomatic patients remains unclear. Provided that the risk of events in BrS is not truly stochastic, distinct sub-phenotypes of the condition, possessing variable levels of arrhythmic risk, may exist, and their identification may lead to the improved care of BrS patients and their families.

## Introduction

Brugada syndrome (BrS) is a rare inherited arrhythmia disorder that is associated with sudden cardiac death secondary to malignant ventricular arrhythmias.^1^ Its hallmark feature, the type I electrocardiograph (ECG) pattern, is characterized by pseudo-right bundle branch block morphology and coved ST-segment elevation in the right precordial leads.^2^ The global prevalence of the type I ECG pattern has been estimated at 0.05%, although considerable geographic variability does exist, for example with higher frequencies observed in Asia.^3^ Since its original description approximately 25 years ago, significant progress has been made towards unraveling BrS’s mechanistic underpinnings, including the identification of the 23 genes suggested to predispose individuals to its development.^2,4,5^

Despite having made considerable strides forward in the understanding of the condition, critical gaps continue to persist, making efficient clinical management of affected individuals challenging.^6^ An underlying genetic culprit continues to be elusive in the majority of patients, while discord regarding BrS’s underlying pathophysiology persists, with strong lines of evidence available for both the “depolarization” and “repolarization” hypotheses.^5,7^ For clinicians, a major challenge that remains is the effective risk stratification of asymptomatic patients who, by guidelines, are currently managed with observation.^8^ Although the risk of a BrS-related event for asymptomatic individuals is low, and currently outweighed by the hazards associated with the insertion of a transvenous implantable cardioverter-defibrillator (ICD), sudden cardiac death (SCD) in young, otherwise healthy individuals is catastrophic and rightfully so considered to be an unacceptable outcome. This conundrum highlights the need for novel strategies for risk stratification and safer, more effective treatment options. Our evolving insights into the genetics and pathophysiology underlying BrS may facilitate identification of atrisk “sub-phenotypes,” while subcutaneous ICDs (S-ICDs) and catheter ablation are promising new forms of therapy that may lead to improved patient outcomes.^9,10^

### Genetic contributions

The recognition of familial clustering in BrS highlighted its being a heritable condition, and ultimately led to the identification of *SCN5A* as the first genetic culprit, via a candidate gene approach.^11^ *>SCN5A* encodes the α-subunit of the cardiac voltage-gated sodium channel (Nav1.5), which is responsible for the inward sodium current (*I*_Na_).^12^ This seminal finding provided critical mechanistic insight into BrS and highlighted reduced *I*_Na_ as being a cornerstone of the condition’s underlying pathophysiology. Since this initial report, over 300 distinct pathogenic loss-of-function *SCN5A* mutations have been implicated in the development of BrS and, collectively, are detected in approximately 20% to 25% of all clinical cases.^13^

Beyond *SCN5A,* an additional 22 genetic culprits have been identified as predisposing a patient to developing BrS **(Table 1)**.^5^ Consistent with BrS’ being reflective of a channelopathy, the majority of the culprits encode ion channels that mediate currents involved in the cardiac action potential. *SCN10A* encodes a neuronal sodium channel, while *SCN1B, SCN2B,* and *SCN3B* encode β-subunits that modulate Nav1.5.^14–17^ Consistent with *SCN5A,* identified mutations have been predicted to result in a loss-of-function and in reduced *I*_Na_. Gain-of-function mutations within *KCNE3, KCNE5, KCND2,* and *KCND3* have been shown to increase *I*_to_ (Phase 1; transient outward current), while loss-of-function mutations within *CACNAlc, CACNB2b,* and *CACNA2D1* reduce *I*_Ca_ (Phase 2; inward calcium current) resulting in an abbreviated plateau of Phase 2 of the action potential have also been identified in BrS patients.^18–21^ *KCNJ8* and *ABCC9* are constituents of the ATP-sensitive potassium current (*I*_K-ATP_), while *KCNH2* is the α-subunit of *I*_Kr_, and increased current secondary to gain-of-function mutations has been suggested to predispose to BrS.^22,23^ The remaining genetic culprits (i.e. *GPD1L, RANGRF, SLMAP, PKP2, FGF12, HEY2, HCN4, TRPM4,* and *SEMA3A),* although not constituents of the ion channels directly implicated in the cardiac action potential, have all been suggested as being involved in predisposing individuals to BrS, secondary to the modulation of one of the ionic currents described above.^5^

Although BrS is traditionally viewed as a monogenic autosomal dominant condition, recent studies have increasingly challenged this notion, and current evidence suggests that the BrS phenotype more often develops secondary to oligo- or polygenic influences. Notably, the only BrS gene identified through linkage analysis has been *GPD1L,* which encodes the glycerol-3-phosphate dehydrogenase 1-like gene that is hypothesized to predispose to BrS through a reduction in *I*_Na_.^24^ Loss-of-function *SCN5A* mutations are likely sufficient in isolation to give rise to the phenotype, though penetrance is highly variable. Even in the context of *SCN5A,* however, additional genetic influences are likely operative, a concept that has been highlighted by a study involving 13 families who possess a presumed pathogenic *SCN5A* mutation.^25^ Notably, the BrS phenotype was observed among individuals from five of these families in the absence of the *SCN5A* mutation.

Beyond *SCN5A* and *GPD1L,* the remaining genetic culprits may be better characterized as disease susceptibility variants, rather than BrS-causing mutations. Each of these additional genes was identified through the candidate gene approach following identification of mutations in a limited number of individuals. Although supportive *in vitro* functional work was often provided, in most instances, the evidence for genotype–phenotype segregation remained lacking. Following the advent of next-generation sequencing and the subsequent establishment of large population-based exome and genome cohorts, it has become evident that the collective prevalence of BrS-linked mutations in these additional genes is much higher than expected for highly penetrant monogenic culprits. Notably, within the United States National Heart, Lung, and Blood Institute’s Grand Opportunity Exome Sequencing Project, one in 23 individuals was found to possess a genetic variant classified as pathogenic for BrS.^26^ These findings serve to emphasize that these genetic variants may predispose certain individuals to BrS; however, additional genetic or environmental influences are likely required for the development of the phenotype.

As further support for a polygenic disease process, a genome-wide association study identified three single-nucleotide polymorphisms in the vicinity of the *SCN5A, SCN10A,* and *HEY2* genes that are associated with an increased risk for developing the condition.^27^ Interestingly, the odds ratios for these variants, ranging from 1.58 to 2.55, align with the notion that though they are predisposing, they are not sufficient in isolation to give rise to a BrS phenotype. Given that BrS is a rare condition, the presumption has been that rare, rather than common, genetic variants are responsible for its development. It is likely then that the development of a BrS phenotype is dependent upon a combination of common and rare variants, accounting for the complex inheritance patterns that are observed in the clinic, and the challenging nature of gene discovery.

### Pathophysiology

Mirroring the genetic landscape and partly guided by its continued progress, major strides have been made towards clarifying BrS pathophysiology, but many questions still remain. Although there is a consensus that BrS’s pathophysiology localizes to the right ventricular outflow tract (RVOT), there is still some debate that continues to persist regarding whether BrS is a disorder of depolarization, repolarization, or both.^28^ The repolarization hypothesis posits that the arrhythmogenic substrate develops secondary to either a pathologic reduction in *I*_Na_, an increase in *I*_to_, or both.^29^ *I*_to_, being most prominent on the epicardial surface of the RVOT, is hypothesized to account for its pathophysiology localizing to that region.^30^ The pathologic alteration in either *I*_Na_ or *I*_to_ leads to the loss of the Phase 2 action potential dome within the epicardium; the resultant transmural repolarizing voltage gradient across the RVOT is hypothesized then to account for the characteristic type 1 Brugada ECG pattern.^29,31^ In addition, this transmural dispersion of repolarization is felt to provide a substrate sufficient for phase 2 re-entry, which may subsequently trigger and/or manifest clinically as polymorphic ventricular tachycardia (VT). Notably, a similar mechanism has been hypothesized to be responsible for the early repolarization syndrome, leading investigators to collectively refer to both conditions as J-wave syndromes.^32^

The other primary competing hypothesis contends that the BrS phenotype develops secondary to a depolarization abnormality associated with conduction slowing within the RVOT.^33^ The proposed mechanism of arrhythmogenesis is the production of a gradient between the RVOT and the adjacent right ventricular (RV) myocardium secondary to this conduction delay, and potentially secondary to fibrosis. Support for the depolarization hypothesis has been bolstered by intriguing findings from Nadamanee et al.,^10^ who identified low-voltage regions along the anterior aspect of the epicardial RVOT that possessed late potentials and fractionated electrograms in BrS patients. Perhaps most strikingly, ablation of these signals rendered ventricular fibrillation non-inducible with programmed extra-stimulation, resulted in the normalization of the type 1 Brugada ECG pattern, and led to the effective clinical suppression of arrhythmias during long-term follow-up. Subsequent investigations performed on autopsies of whole hearts, as well as biopsies obtained at the time of ablation via thoracotomy, revealed that the RVOT of BrS patients had increased collagen deposition and fibrosis, coupled with reduced connexin-43 expression compared with the controls.^34^

Although in apparent direct opposition to one another, it is possible that both depolarization and repolarization abnormalities may be operative in BrS pathogenesis. Utilizing non-invasive ECG imaging (ECGI), a technology that records surface ECG potentials using 250 electrodes, investigators identified delayed RVOT activation, along with low amplitude and fractionated electrograms, in BrS patients, which are findings consistent with a depolarization abnormality.^35^ Concurrently, however, the patients included in the study were also observed to have prolonged recovery times and sleep repolarization gradients, leading the authors to conclude that abnormalities in both depolarization and repolarization contribute to the BrS phenotype.

### Diagnosis

**Figure 1:**
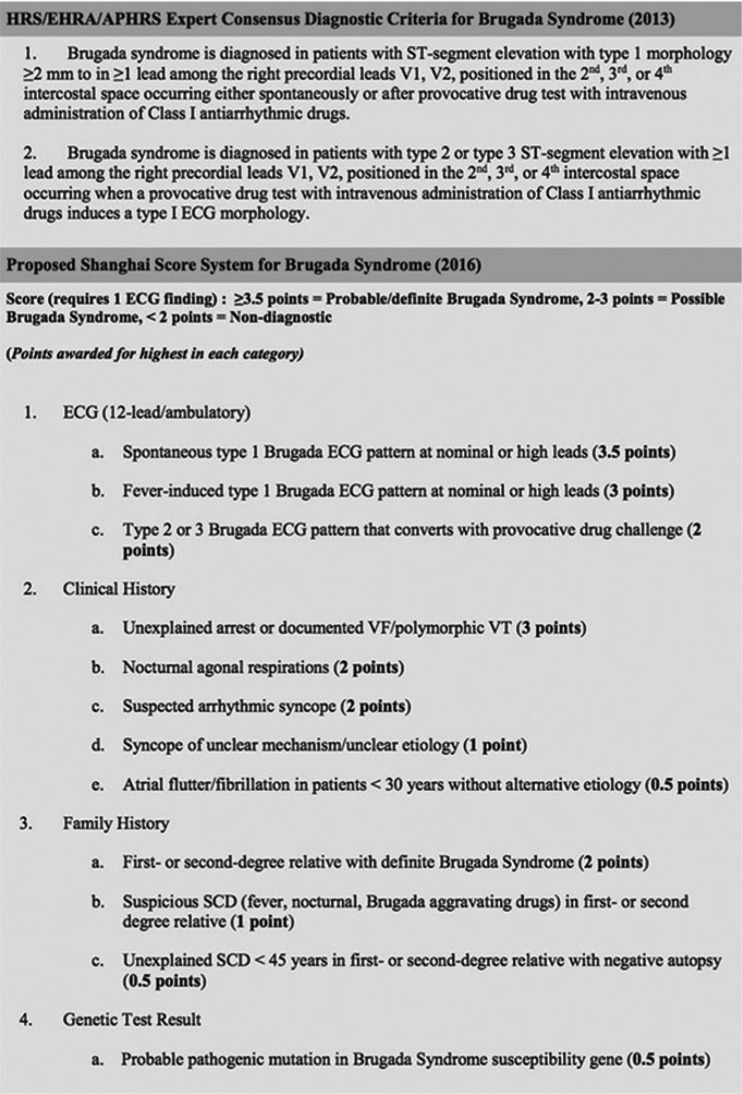
Alternate contemporary criteria for the diagnosis of Brugada syndrome. (a) HRS/EHRA/APHRS Expert Consensus Diagnostic Criteria for the Brugada Syndrome; (b) Shanghai Score System for Diagnosis of Brugada Syndrome. HRS: Heart Rhythm Society; EHRA: European Heart Rhythm Association; APHRS: Asia Pacific Heart Rhythm Society; VF: ventricular fibrillation; VT: ventricular tachycardia; SCD: sudden cardiac death.

The criteria for diagnosing BrS have evolved since the condition’s initial description, and debate continues regarding the need for additional identifiable clinical features beyond the distinctive electrocardiographic pattern, particularly in cases in which a type 1 pattern is only observed during provocative drug challenge. Criteria for concluding a type 1 ECG pattern require J-point elevation ≥ 2 mm in one or more lead among V1 or V2 positioned in the second, third, or fourth intercostal space, in association with a coved ST-segment morphology, whereas the type 2 pattern requires ≥ 2 mm of J-point elevation in similar lead positions in association with a saddleback ST-segment morphology.^36^ The most recent Heart Rhythm Society/European Heart Rhythm Association/Asia Pacific Heart Rhythm Society (HRS/EHRA/APHRS) expert consensus statement indicates that a type 1 Brugada ECG pattern, either spontaneous, fever, or drug induced, is sufficient to satisfy a diagnosis of BrS **(Figure 1a)**.^8^

Recent studies have highlighted concern regarding the possibility of a high false-positive rate with provocative drug challenge, particularly in the case of ajmaline use, although the lack of a gold standard renders this concern challenging to assess in an objective manner.^37,38^ Driven by concern for over-diagnosis, experts have proposed the Shanghai Score System that, beyond the ECG analysis, also accounts for clinical and family history and genetic testing results **(Figure 1b)**.^39^ The distinctive feature of this scoring system, relative to the recent HRS/EHRA/APHRS expert consensus statement, is the requirement for additional features to be considered beyond the ECG in order to conclude a probable/definite BrS diagnosis in cases in which a type 1 pattern is exclusively observed during fever or a provocative drug challenge.

It should also be noted that a type 1 Brugada ECG may also be provoked by a variety of clinical insults and conditions, including myocardial ischemia, metabolic abnormalities, and pectus excavatum. In cases in which the ECG normalizes following removal of the clinical insult or condition, a subsequent provocative drug fails to induce a type 1 pattern and/or genetic testing is negative, experts have suggested to label these cases as Brugada phenocopies.^40^ The rationale for this alternative terminology is as an attempt to differentiate these cases from typical BrS, as they are presumed to have a non-genetic etiology and carry a more benign prognostic significance. This nomenclature remains controversial, however, as our limited understanding of the genetic culprits of BrS limits our ability to exclude an underlying genetic predisposition.

### Treatment

Current therapeutic options are relatively limited for BrS, with ICD therapy being the only proven treatment strategy for the prevention of SCD.^8^ Expert consensus guidelines currently recommend ICD therapy as a class 1 indication for patients with prior cardiac arrest or documented polymorphic VT, whereas a spontaneous type 1 ECG pattern with a history suggesting arrhythmic syncope is a class IIa indication.^8^ Although effective for preventing SCD, ICD implantation also carries a significant risk of complications over the patient’s lifetime, particularly if the patient is younger at the time of insertion. Beyond a high prevalence of inappropriate shocks, ICD implantation at a young age also exposes patients to recurrent risks of infection secondary to pulse generator changes and inevitable lead complications that often necessitate subsequent extraction procedures that carry a risk of death.^41^

The S-ICD is a new treatment option that appears to be ideally suited for patients with BrS given its ability to avoid the pitfalls associated with long-term use of transvenous lead systems, coupled with the fact that intracardiac pacing is rarely required in BrS patients.^9^ It is conceivable that the alternate benefit–risk profile of the S-ICD may permit a more aggressive approach to management in asymptomatic patients with high risk features who are currently being managed conservatively, owing to concerns for adverse events associated with transvenous devices. Although avoiding a transvenous device is appealing, it should be noted that there may be a comparable risk of non-lead-related complications over a lifetime.^42^ As such, larger studies with longer-term follow-up will be necessary to further clarify relative benefits of S-ICD therapy in this patient population. One important consideration for S-ICD use in BrS patients is the potential need to perform vector testing to evaluate the patient’s suitability for implantation during drug challenge, when the type 1 ECG pattern is transient.

This is because the development of a type 1 Brugada pattern can significantly change the sensing vectors, rendering the patient a poor candidate for S-ICD therapy secondary to an increased risk for T-wave oversensing. This is particularly relevant given that arrhythmic risk is only felt to develop when the type 1 pattern emerges. A recent analysis of 21 patients who underwent S-ICD implantation found that morphology analysis failed in 24% of the patients following development of the type 1 Brugada pattern.^43^

Chronic medical therapy for BrS is generally reserved for individuals who suffer recurrent ICD shocks. Quinidine is the treatment of choice, and its efficacy is presumed to be secondary to its I_to_-blocking activity.^30^ Multiple reports have highlighted quinidine’s ability to effectively suppress ventricular arrhythmias and, although breakthroughs have been reported, they are considered rare.^44^ As an alternative approach for management, Belhassen and colleagues advocate for electrophysiologically guided quinidine therapy, in which all patients undergo an invasive electrophysiology study, and those in whom ventricular fibrillation is inducible with programmed extra-stimulation are subsequently initiated on the medication.^45,46^ A second invasive electrophysiology study is then performed to confirm that ventricular fibrillation is no longer inducible, a goal that is typically achieved in 90% of patients. Utilizing this approach, they had no treatment failures among the 96 patients followed for a mean of 113 months.

Catheter ablation has also recently emerged as a compelling treatment option for BrS, particularly given its perceived potential to serve as a curative form of therapy.^10^ Initial attempts to treat BrS with catheter ablation have been reported by Haissaguerre and colleagues,^47^ who targeted triggers in the form of premature ventricular contractions from the RVOT endocardium. Although effective in a limited number of patients, the efficacy of the approach is limited by the fact that triggers in BrS are rarely observed. The substrate-based approach reported by Nademanee in 2011 instead targeted fractionated and late potentials identified along the RVOT epicardium for ablation, obviating the need for identifying triggers. Strikingly, in this study, the substrate-based approach led to the normalization of the surface ECG and the cessation of arrhythmic events among BrS patients who had been suffering from recurrent ICD shocks refractory to medical therapy. Since this initial report, similar results have been obtained in other, larger studies with longer follow-up.^34,48^

Although the results of substrate-based catheter ablation have been extremely promising, arrhythmic recurrences have been reported despite normalization of the ECG.^49^ Notably, it appears that although the type 1 ECG pattern was no longer present spontaneously in these patients, it could still be induced with drug provocation. This realization led to the mapping of the RVOT epicardium in the presence of sodium channel blockade, which resulted in the identification of larger regions with fractionated and late potentials that could be targeted with ablation.^50^ This approach, which appears to permit for a more complete identification of the BrS arrhythmogenic substrate, has resulted in improved long-term clinical results, and will hopefully be sufficient to overcome prior treatment failures.^48^ In order to further clarify the efficacy of catheter ablation, a randomized controlled trial, called the Ablation in Brugada Syndrome for Prevention of VF (BRAVE) study, is being initiated.^51^ BrS patients who have suffered an ICD shock will be randomized to either receive catheter ablation or no additional therapy, and will be followed for up to three years for recurrent malignant arrhythmias.

### Arrhythmic risk

Insight into the risk of arrhythmic events among individuals with a type 1 Brugada pattern has markedly evolved over the last two decades. The initial report from the Brugada group in 1998 reported a 32% incidence for ventricular fibrillation and sudden cardiac death during a 3-year follow-up period.^52^ Reflecting a pattern observed in many newly discovered syndromes, the initially reported alarmingly high event rate is now felt to have been likely secondary to the cohort being comprised of cases with more severe phenotypes. Following improved recognition of BrS among physicians and the establishment of registries that include a broader spectrum of phenotypic severities, it has become apparent that the risk of events in asymptomatic individuals is in fact quite low, accounting for the recommendations regarding management being restricted to observation in the majority of these individuals.

The FINGER Brugada Syndrome Registry was a multicenter study involving 1,029 European patients who exhibited spontaneous or drug-induced type 1 ECG patterns with a mean follow-up period of 32 months.^53^ In this study, the annual cardiac event rate identified was highest among those with a history of aborted cardiac arrest (7.7%), while patients with a history of presumed cardiac syncope and asymptomatic patients had event rates of 1.9% and 0.5%, respectively. Comparable findings were also observed in the PRELUDE study, which prospectively followed 308 BrS patients with no prior history of cardiac arrest.^54^ An overall annual event rate of 1.5% was observed in this study during a mean follow-up period of 36 months, which corresponded to event rates of 3.6% and 1.0% among patients with prior instances of cardiac syncope and asymptomatic individuals, respectively. A more recent report from the Brugada group has revealed similar findings, with a notable temporal dichotomy present within their cohort among individuals enrolled from 1986 to 2002 and from 2003 to 2014.^55^ The annual event rate was 2.5% in the earlier cohort, as compared with 1.8% in the later cohort (p<0.001). Notably, asymptomatic patients with a drug-induced type 1 pattern had a 0.51% annual event rate, consistent with findings from other contemporary studies.

### Risk stratification in asymptomatic individuals

Although ICD therapy is justified for individuals with episodes of prior aborted cardiac arrest or cardiac syncope given their high risk for fatal arrhythmias, asymptomatic patients have a low incidence of events, rendering treatment challenging given the risks associated with long-term ICD therapy. Widespread ICD implantation in asymptomatic BrS patients has had an unfavorable risk–benefit profile; however, a watchful waiting strategy is inevitably expected to witness incident cases of SCD, an occurrence that is unacceptable in young, otherwise healthy individuals. This conundrum highlights a critical need to develop more effective risk stratification tools in asymptomatic individuals to facilitate targeted ICD therapy in the small minority of individuals who will succumb to SCD during follow-up.

Various clinical features have been evaluated in asymptomatic BrS patients in an attempt to identify those at increased risk for malignant arrhythmic events, including age, sex, and family history of SCD. Mounting evidence suggests that asymptomatic BrS patients >60 years of age, and particularly those >70 years of age, have very low event rates, implying that ICD therapy may be largely unnecessary in this elderly patient subgroup.^56^ Although BrS is more prevalent among males, perhaps secondary to the influence of testosterone on *I*_to_, a reduced risk of arrhythmic events in females has not yet been clearly established.^53^ Similarly, a positive family history of SCD has not yet been shown to confer a worse prognosis for BrS patients.^53^ The role of genetic testing for informing prognosis has also been evaluated and, and, although mixed results have been observed, there is no strong evidence to date to suggest that genotype can serve as a reliable predictor of arrhythmic events.^53^

### ECG features

**Figure 2:**
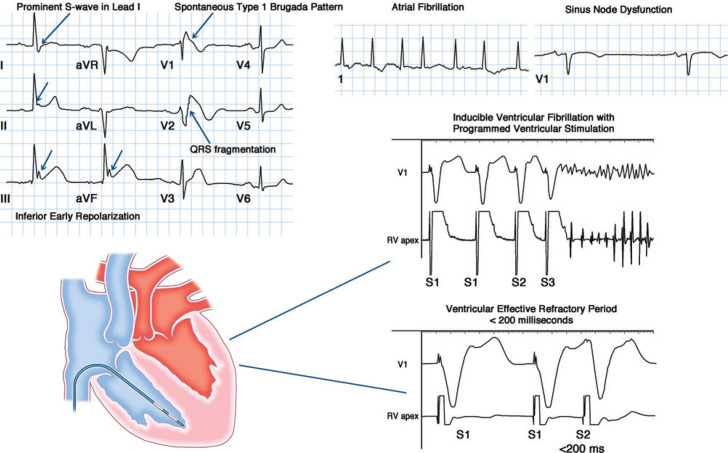
Clinical, electrocardiographic, and electrophysiologic modifiers of arrhythmic risk in patients with Brugada syndrome.

A series of surface ECG features **(Figure 2)** has been suggested to predict an increased risk of arrhythmic events in BrS, including QRS fragmentation, early repolarization and/or a prominent S-wave in lead I. QRS fragmentation, referring to multiple sharp deflections observed during depolarization, has been variably defined in the BrS literature based on the number of “spikes” observed.^54,57^ Despite the different definitions, however, multiple studies have identified an association between QRS fragmentation and a risk of incident events. When prospectively evaluated in the PRELUDE registry and defined as ≥ two spikes within the QRS complex in leads V1 to V3, the presence of QRS fragmentation was associated with a statistically significant 8.9-fold increased hazard of arrhythmia.^54^

The S-wave in lead I, partially mediated by depolarization of the RVOT, has recently been suggested to serve as a powerful marker for arrhythmic risk in BrS.^58^ Among 347 asymptomatic patients with a spontaneous type 1 Brugada pattern, the presence of a significant S-wave in lead I, defined as ≥0.1 mV and/or ≥40 ms, was associated with a staggering 39.1-fold increased hazard of ventricular fibrillation or SCD on multivariate analysis.^58^ Although this finding will need to be replicated in additional studies, the magnitude of association observed suggests it could serve as a critical tool for risk stratification. Mirroring numerous other cardiac conditions, the presence of an inferolateral early repolarization pattern has been shown to confer an increased risk of events among BrS patients.^59,60^ This finding has been replicated in multiple studies and has been further reinforced by a recent meta-analysis, consistent with its being a reliable marker of arrhythmic risk.^61^ The concomitant presence of atrial arrhythmias, including sinus node dysfunction and atrial fibrillation, has also been suggested to confer an increased risk of malignant arrhythmic events.^62,63^

### Electrophysiology study

The role of invasive electrophysiology study in identifying BrS has been controversial, with disagreement regarding its clinical utility, a notion currently reflected in that it has a class IIb indication as a tool for risk stratification.^54^ Debate surrounding its utility partially stems from variable results having been described in the literature, with certain reports from the Brugada group suggesting that it is highly predictive of events, although other studies have found no association.^64,65^ Reasons for the conflicting results have been hypothesized to be secondary to both heterogeneous patient populations having been evaluated and variations being present within the programmed extra-stimulation protocols used for the induction of polymorphic ventricular tachycardia/ventricular fibrillation.

In an effort to provide clarity, Priori and colleagues^54^ conducted the PRELUDE study, a multicenter prospective registry involving 308 BrS patients with no prior history of SCD. A pre-specified induction protocol was used consisting of 600 and 400 ms drive trains with the addition of up to three extra-stimuli from both the RVOT and RV apex. Consistent with their prior work, the investigators found no association between inducibility at electrophysiology study and subsequent arrhythmic events during a mean follow-up period of 36 months. The sensitivity and specificity of programmed extra-stimulation for predicting subsequent arrhythmic events with up to three extra-stimuli was determined to be 35.7% and 58.8%, respectively. Limiting programmed extra-stimulation to one to two extra-stimuli improved specificity to 74.2%, but lowered sensitivity to 25%. Highlighting additional concern for reproducibility of the test, only 34% of inducible patients could be re-induced at the time of a second electrophysiology study. Although ventricular fibrillation inducibility was not predictive of events, a large magnitude association was observed between a ventricular refractory period <200 ms and subsequent arrhythmic events (hazard ratio = 3.91, 95% CI 1.03–12.79) **(Figure 2)**.^54^

A subsequent pooled analysis with individual-level data that combined eight prior studies and a total of 1,312 patients, though not involving prior work from the Brugada group, found that inducibility at the time of invasive electrophysiology study was associated with an increased risk of events, with a hazard ratio of 2.66 (95% CI 1.44–4.92) and a higher risk observed among those induced with single or double extra-stimuli.^66^ Perhaps equally important, however, was that the lack of induction exhibited a modest negative predictive value, leading the authors to caution that a negative electro-physiology study should not be used as evidence to avoid ICD implantation, especially when measured in relation to other high-risk features like a spontaneous type 1 pattern or syncope presumed to be cardiac in origin. These important findings have led to a resurgence of interest in the role of invasive electrophysiology study as a tool for risk stratification in BrS.

### Multivariable risk prediction model

Consistent with most medical conditions, it is unlikely that a single factor in isolation will be sufficient as a means for accurately predicting arrhythmic risk in asymptomatic BrS individuals. Composite risk scores that account for multiple variables may ultimately yield more accurate measures of risk prediction, in a manner similar to that of the Framingham Risk Score for coronary artery disease. A series of studies has evaluated this concept in BrS and suggested promising results, though their relatively small size, compounded by the low observed event rates, highlight the need for larger scale studies prior to incorporation of such models into widespread clinical practice.^67,68^

## Conclusions

Since its original description approximately 25 years ago, major strides have been made towards unraveling the mechanisms underlying BrS. In a similar manner, our treatment of BrS patients has progressively evolved during this period, with gradually improved insight into the benefits and risks of different treatment strategies. Exciting new therapeutic options hold significant promise, though deciding when and how to intervene in asymptomatic patients will likely remain a vexing challenge. Provided that the risk of events in BrS is not truly stochastic, distinct sub-phenotypes of the condition possessing variable levels of arrhythmic risk are presumably operative. It is hoped that our rapidly progressing knowledge of the genetic and pathophysiologic underpinnings may yield insight into the existence of these sub-phenotypes, leading to improved care of BrS patients and their families.

## Figures and Tables

**Table 1:** Genetic Culprits Implicated in Brugada Syndrome

Gene	Protein	Impact on Ionic Current
*SCN5A*	α-subunit of Nav1.5	↓/_Na_
*GPD1L*	Glycerol-3-phosphate dehydrogenase 1-Like	↓/_Na_
*CACNA1C*	α-subunit of Cav1.2	↓/_ca_
*CACNB2b*	β-subunit; Cavβ2	↓/_ca_
*SCN1B*	β-subunit; Navβ1	↓/_Na_
*KCNE3*	β-subunit of potassium channel (MiRP2)	↑/_to_
*SCN3B*	β-subunit; Navβ3	↓/_Na_
*HCN4*	Hyperpolarization-activated cyclic nucleotide-gated channel 4	*
*KCND3*	α-subunit of Kv4.3	↑/_to_
*KCNJ8*	α-subunit of Kir6.1	↑/_K-ATP_
*CACNA2D1*	δ-subunit of Cavα2δ1	↓/_Ca-L_
*KCNE5*	β-subunit of potassium channel	↑/_to_
*RANGRF*	RAN guanine nucleotide release factor	↓/_Na_
*KCND2*	α-subunit of Kv4.2	↑/_to_
*TRPM4*	Transient receptor potential cation channel subfamily M member 4	*
*SCN2B*	β-subunit; Navβ2	↓/_Na_
*PKP2*	Plakophilin-2	↓/_Na_
*ABCC9*	Sulfonylurea receptor-2	↑/_K-ATP_
*SLMAP*	Sarcolemmal membrane-associated protein	↓/_Na_
*KCNH2*	α-subunit of HERG	T/Kr
*SCN10A*	α-subunit of Nav1.8	↓/_Na_
*FGF12*	Fibroblast growth factor-12	↓/_Na_
*SEMA3A*	Semaphorin-3A	↑/_to_

*The impact on ionic current is not well-established for *HCN4* and *TRPM4* mutations.
